# Cytoreduction plus hyperthermic intraperitoneal chemotherapy for peritoneal carcinomatosis in colorectal cancer patients: a single-center cohort study

**DOI:** 10.1186/s12957-019-1602-z

**Published:** 2019-03-27

**Authors:** Leonardo Solaini, Fabrizio D’Acapito, Alessandro Passardi, Massimo Framarini, Francesca Tauceri, Daniela Di Pietrantonio, Giovanni Luca Frassineti, Andrea Casadei Gardini, Alessandro Cucchetti, Davide Cavaliere, Giorgio Ercolani

**Affiliations:** 1 General and Oncologic Surgery, Morgagni-Pierantoni Hospital, AUSL Romagna, via Forlanini 34, 47121 Forlì, Italy; 2 Department of Medical and Surgical Sciences, University of Bologna, via Zamboni 33, 40126 Bologna, Italy; 3 Department of Medical Oncology, Istituto Scientifico Romagnolo per lo Studio e Cura dei Tumori (IRST) IRCCS, via Maroncelli 40, 47014 Meldola, Italy

**Keywords:** Hyperthermic intraperitoneal chemotherapy, Peritoneal carcinomatosis, Colorectal cancer, Cytoreduction, Outcome

## Abstract

**Background:**

In this study, we report our experience of cytoreductive surgery plus hyperthermic intraperitoneal chemotherapy (CRS-HIPEC) in patients with peritoneal carcinomatosis (PC) from colorectal cancer (CRC), focusing on the factors affecting survival.

**Methods:**

All patients with surgically treated PC from colorectal cancer and with no involvement of other organs referred to our institute from March 2005 to December 2017 were included in the analysis.

**Results:**

Thirty-eight patients underwent CRS-HIPEC, and all had a completeness of cytoreduction score of 0 (CC0). The median operating time was 645 min (interquartile range [IQR] 565–710). Five patients (13.1%) had Clavien-Dindo grade > 2 postoperative complications. Median overall survival (OS) was 60 months. In the Cox regression for OS, calculated on the CRS-HIPEC group, the peritoneal cancer index (PCI) > 6 (hazard ratio [HR] 4.48, IQR 1.68–11.9, *P* = 0.003) and significant nodal involvement (N2) (HR 3.89, IQR 1.50–10.1, *P* = 0.005) were independent prognostic factors. Median disease-free survival (DFS) was 16 months. Only N2 (HR 2.44, IQR 1.11–5.36, *P* = 0.027) was a significantly negative prognostic factor for DFS in multivariate analysis.

**Conclusions:**

CRS-HIPEC can substantially improve survival. However, patients with high PCI (PCI > 6) and significant nodal involvement (N2) may not benefit from the procedure.

## Introduction

The peritoneum is the second most common site of metastasis in colorectal cancer (CRC), with a incidence of 25–35% [1, 2]. Peritoneal carcinomatosis (PC) confers a poorer prognosis than other metastatic sites [3, 4]. It has been seen that patients with PC undergoing modern systemic chemotherapy show a median survival of at least 22 months [3]. Cytoreduction plus hyperthermic intraperitoneal chemotherapy (CRS-HIPEC) [5, 6] represents a further treatment option for patients with PC from CRC, obtaining median survival rates of around 60 months [7–10]. However, studies on CRS-HIPEC are characterized by limited sample sizes, heterogeneous patients, and lack of control groups, resulting in guidelines containing weak recommendations based on low-quality evidence [11].

In the present study, we analyze our experience of CRS-HIPEC in patients with PC from CRC, focusing on the factors influencing survival.

## Material and methods

Patients with surgically treated PC from CRC without involvement of other organs referred to our institute from March 2005 to December 2017 were included in this analysis. Exclusion criteria were age > 75 years, American Society of Anesthesiologists (ASA) score > 2, and technically unresectable tumor (massive involvement of hepatic hilum or full-thickness involvement of the diaphragm). The work was reported in line with the Strengthening the Reporting of Cohort Studies in Surgery (STROCSS) criteria [12].

**Table 1:** Patients’ characteristics

Variables	
Age, years—median (IQR)	61 (53–70)
Female, *n* (%)	20 (52.6)
Site of the primary tumor	
Rectum	5 (13.2)
Right colon	14 (36.8)
Transverse and sigmoid colon	19 (50.0)
Grade ≥ 3, *n* (%)	16 (42.1)
T3–T4, *n* (%)	37 (97.4)
N+, *n* (%)	25 (65.8)
Synchronous carcinomatosis, *n* (%)	14 (36.8)
Peritoneal cancer index—median (IQR)	5 (2–9)

### Definitions and treatment protocol

The primary outcome measure was OS which was defined as the time between surgery and last follow-up or death. DFS was defined as shown elsewhere [13]. The definitions of peritoneal cancer index (PCI) and completeness of cytoreduction (CC) score are defined in detail elsewhere [14]. CC0 defines no residual peritoneal lesions within the operative field, CC1 refers to persisting nodules < 2.5 cm after CRS, CC2 indicates nodules between 2.5 and 5 cm, and CC-3 refers to nodules > 5 cm or confluent unresectable tumor nodules. The 7^th^ edition of the AJCC Cancer Staging Manual was used to stage the disease at histological evaluation [15].

All patients underwent CRS-HIPEC in the Department of General and Oncologic Surgery of Morgagni-Pierantoni Hospital, Forlì (Italy). As per protocol, HIPEC was offered to ASA 1-2 patients > 75 years. Peritonectomies were performed using the Sugarbaker technique [6]. Peritoneal washings were performed with intravenous 5-fluorouracil (400 mg/m^2^) and intraperitoneal oxaliplatin (400 mg/m^2^) for 30 min at 41.5 °C.

### Statistical analysis

An additional analysis was performed comparing patients receiving CRS-HIPEC at our center (tertiary referral hospital) with those who had undergone incomplete cytoreduction (no HIPEC) elsewhere before being referred to our oncology unit. Consequently, information regarding operative/early postoperative outcomes and the CC score in the latter group was not available. Median and interquartile range (IQR) were used to present continuous variables which were compared using the Mann-Whitney *U* test. Fisher’s exact test was performed to compare dichotomous variables. The Kaplan-Meier function was exploited for survival analyses, and differences were evaluated by the log rank test. Cox proportional hazard regression was used to identify independent prognostic factors among the variables included in the analyses (age, sex, site of primitive tumor, PCI, synchronous vs metachronous carcinomatosis, pre-post surgery chemotherapy, synchronous vs metachronous HIPEC, tumor grade, tumor [T], and nodal [N] status). The cutoff for PCI used in the survival analyses was calculated with “Cutoff Finder,” as proposed by Budczies et al. [16]. Follow-up time was calculated using the method proposed by Schemper and Smith [17]. MedCalc (MedCalc ® for Windows ® , version 10.2.0.0; MedCalc Software, Ostend, Belgium) was used to perform the statistical analyses.

## Results

Of the 38 patients included in the analysis (Table 1), 25 (65.8%) had undergone preoperative chemotherapy. Surgery in all 38 patients was CC0. Median operating time for CRS-HIPEC was 645 min (IQR 565–710). In the 10 (26.3%) patients treated for synchronous carcinomatosis, resections included 2 right colectomy only, 2 plus rectal resection, 1 plus jejunal resection, 1 plus hysteroannessiectomy, 2 rectal resection only, 1 plus jejunal resection, and 1 left colectomy. Five (13.1%) patients had Clavien-Dindo grade > 2 postoperative complications, of whom three required surgical management (one for evisceration, one for ileal perforation, and one for ileocolic anastomosis leak). There were no cases of in-hospital mortality. Median length of hospital stay was 16 days (IQR 14–20).

**Fig. 1:**
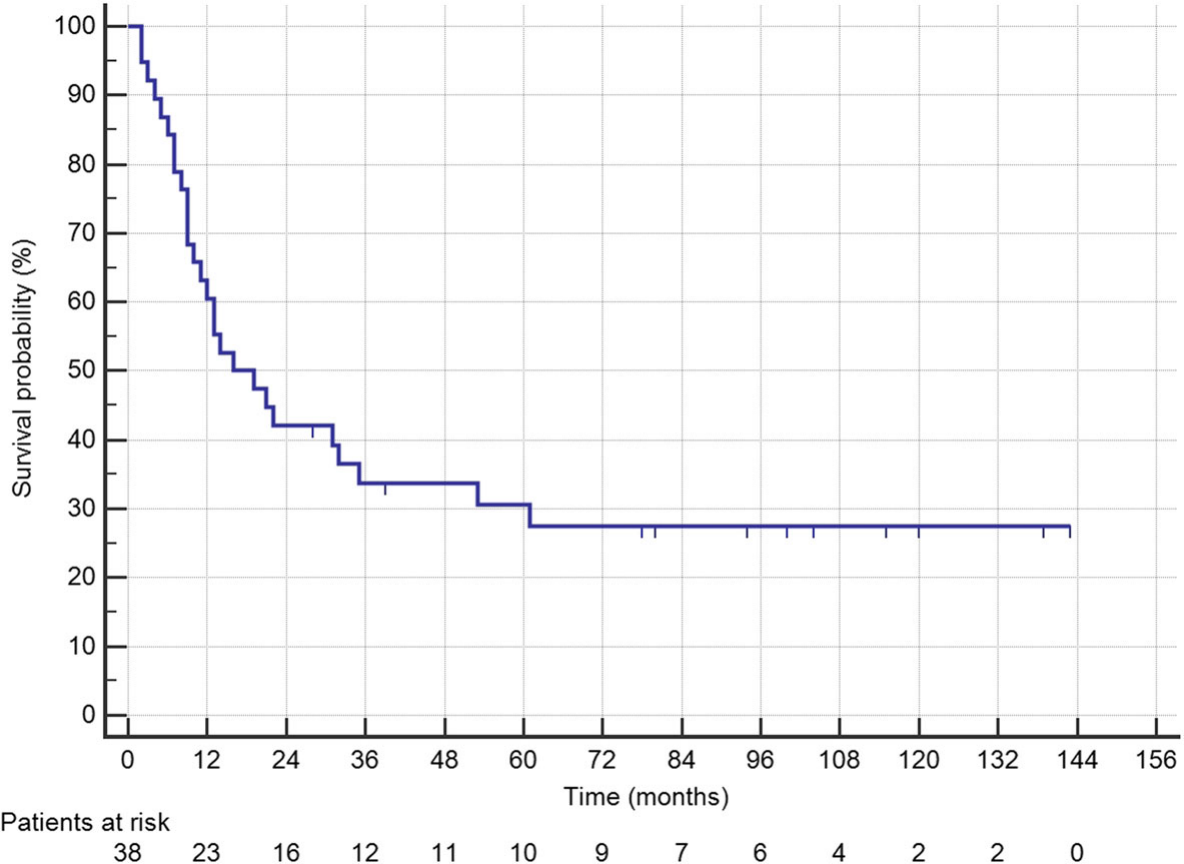
Kaplan-Meier curves showing the disease-free survival of the CRS-HIPEC group

**Fig. 2:**
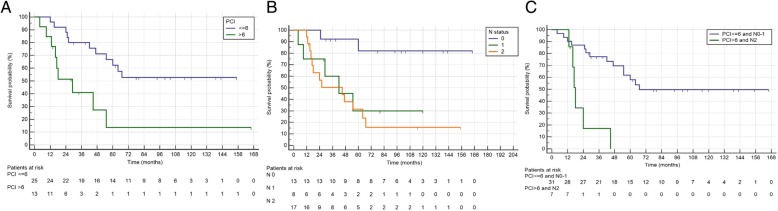
Kaplan-Meier showing survival curves according to PCI (**a**), N status (**b**), and the combination of PCI and N status (**c**)

**Fig. 3:**
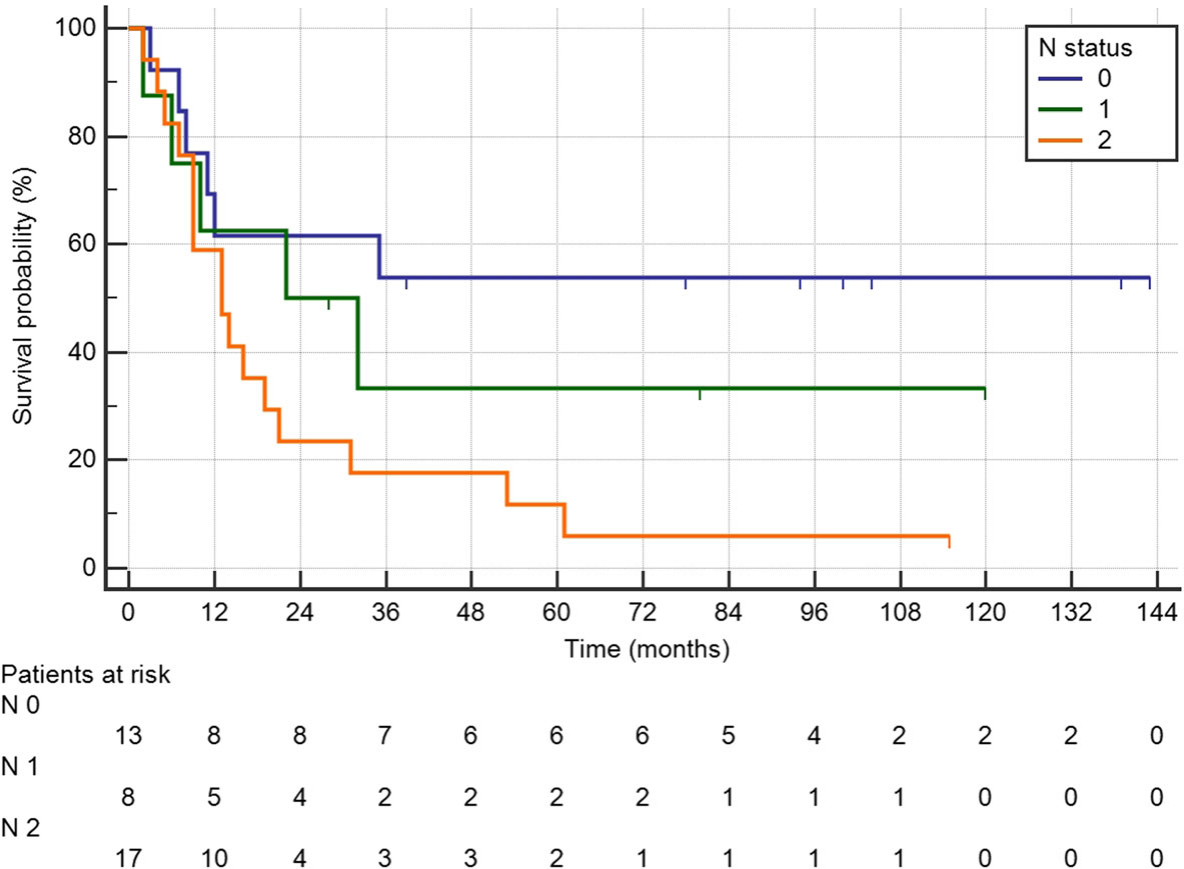
Kaplan-Meier curves showing disease-free survival according to N status (N0 vs N1 vs N2)

Median OS was 60 months, and median DFS was 16 months (Fig. 1). Median follow-up was 115 months (IQR 72–149). In Cox regression for OS, PCI > 6 (HR 4.48 IQR 1.68–11.9, *P* = 0.003) and N2 (HR 3.89 IQR 1.50–10.1, *P* = 0.005) were independent prognostic factors (Table 2). Kaplan-Meier curves of differences in survival according to PCI and N status are shown in Fig. 2a, b. The HR of N2 patients with PCI > 6 was 6.82 (IQR 2.23–20.9, *P* = 0.0008) (Fig. 2c). Only N2 (HR 2.44 IQR 1.11–5.36, *P* = 0.027) was a significantly negative prognostic factor for DFS in multivariate analysis (Table 2) (Fig. 3). Twenty-seven (71%) patients relapsed during follow-up, 16 with multiple sites of recurrence. Twenty-two (81.5%) had peritoneal recurrence and 2 of these underwent a second CRS-HIPEC.

**Table 2:** Cox regression analysis on overall survival and disease-free survival on CC0 patients

Variables	OS				DFS			
	Univariate		Multivariate		Univariate		Multivariate	
	HR (95% CI)	*P*	HR (95% CI)	*P*	HR (95% CI)	*P*	HR (95% CI)	*P*
Age	1.01 (0.96–1.05)	0.737			1.00 (0.97–1.04)	0.705		
Sex (F)	0.77 (0.31–1.83)	0.764			1.20 (0.56–2.56)	0.638		
Right colon cancer	2.51 (0.99–6.31)	0.051			2.37 (1.08–5.23)	0.032		
PCI > 6	3.57 (1.43–8.92)	0.007	4.48 (1.68–11.9)	0.003	2.87 (1.27–6.45)	0.011		
Syncronous carcinomatosis	0.82 (0.34–1.96)	0.652			0.71 (0.33–1.51)	0.380		
Preoperative chemotherapy	1.49 (0.57–3.88)	0.415			1.45 (0.63–3.34)	0.377		
Postoperative chemotherapy	1.59 (0.37–6.88)	0.530			2.71 (0.64–11.5)	0.176		
Syncronous HIPEC	0.50 (0.15–1.70)	0.270			0.49 (0.18–1.28)	0.149		
Postoperative complications Clavien-Dindo > 2	0.33 (0.44–2.45)	0.279			0.48 (0.11–2.04)	0.325		
Tumor grade								
2	1.07 (0.14–8.30)	0.947			1.89 (0.25–14.4)	0.534		
3	1.21 (0.15–9.50)	0.854			2.23 (0.29–16.9)	0.440		
T4 stage	1.13 (0.46–2.76)	0.796			1.04 (0.48–2.26)	0.916		
N stage								
1	6.43 (1.25–33.0)	0.026			1.63 (0.49–5.33)	0.421		
2	8.23 (1.86–36.4)	0.005	3.89 (1.50–10.1)	0.005	3.05 (1.78–7.88)	0.022	2.44 (1.11–5.36)	0.027

### CRS-HIPEC vs incomplete cytoreduction

**Fig. 4:**
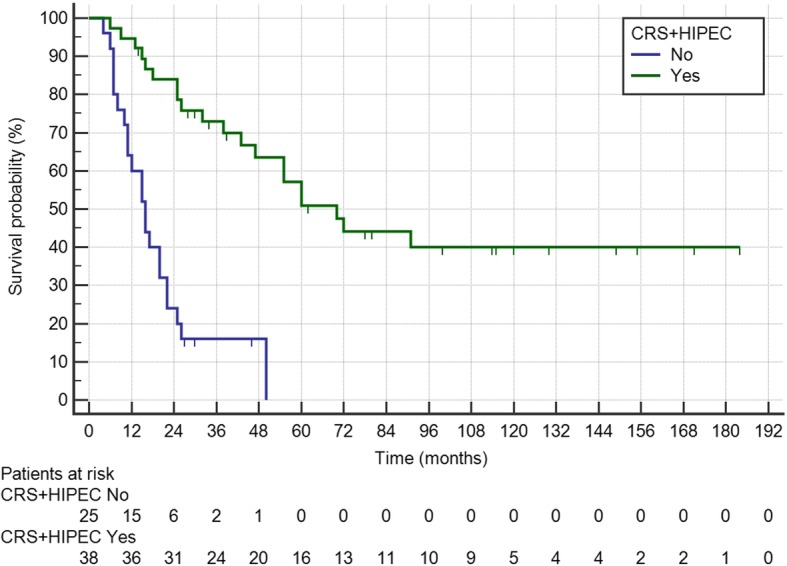
Kaplan-Meier curves showing survival according to the type of treatment received (CRS-HIPEC vs incomplete cytoreduction)

An additional analysis was performed comparing patients submitted to CRS-HIPEC at our center (tertiary referral hospital) (*n* = 38) with those who had undergone incomplete cytoreduction and no HIPEC before being referred to our oncology unit (*n* = 25). The CRS-HIPEC group was comparable with the incomplete CRS group in terms of age (median 61 years, IQR 53–70 vs 66 years, IQR 58–68, respectively; *P* = 382), rate of female patients (20 [52.6%] vs 14 [56%]; *P* = 0.803), right colon cancer (14 [36.8%] vs 11 [44%]; *P* = 0.607), tumor grade ≥ 3 (16, 42.1% vs 11, 44%; *P* = 1.000), N+ tumors (25 [65.8%] vs 16 [64%]; *P* = 1.000), synchronous carcinomatosis (14 [36.8%] vs 11 [44%]; *P* = 0.607), and median PCI (5 [IQR 2–9] vs 6 [IQR 4–12]; 0.078). The number of patients with T3–T4 CRC was significantly higher in the CRS-HIPEC group (37 [97.4%] vs 20 [80%]; *P* = 0.032). The median number of lymph nodes harvested was 24 (IQR 15–35) in the CRS-HIPEC group compared to 32 (IQR 23–39) in the incomplete CRS group (*P* = 0.097). Postoperative chemotherapy was performed in 32 (84.2%) CRS-HIPEC patients compared to 23 (92%) incomplete CRS patients (*P* = 0.461). Median OS of patients with incomplete CRS (median follow-up 30 months, IQR 27–46) was 16 months, significantly lower than that of patients in the CRS-HIPEC group (Fig. 4).

## Discussion

CRS-HIPEC can substantially improve survival in patients with PC from CRC. In our study, median OS of patients undergoing CRS-HIPEC was 60 months, an outcome similar to that reported by others [7–9, 18]. Recently, a metaanalysis [19] including 3179 patients from 15 controlled studies showed that CRS-HIPEC can significantly prolong survival in selected CRC patients with PC with respect to traditional treatments such as palliative surgery alone or systemic chemotherapy (HR = 2.67, 95% CI 2.21–3.23, *P* < 0.00001). Additionally, authors performing a pooled analysis on outcomes from 76 studies (10,036 patients, 16 controlled studies, and 61 non-controlled studies) showed that the median OS was about 29 months in patients undergoing CRS-HIPEC. Of note, only one randomized [20] controlled trial was published during the 30-year period considered in the metaanalysis. In this trial, Verwaal et al. [20] demonstrated a significantly improved survival in patients undergoing CRS-HIPEC and adjuvant systemic 5-fluorouracil with leucovorin compared to those who received systemic 5-fluorouracil with leucovorin alone. The true benefit of CRS-HIPEC in that study was difficult to interpret as the chemotherapy schemes were outdated compared to current ones. Recently, Cashin et al. [21] designed another randomized controlled trial but were forced to terminate it prematurely because of recruitment difficulties. However, they published results on 48 patients (24 per arm), reporting a significant survival benefit in patients who had CRS-HIPEC compared to those treated with oxaliplatin-based chemotherapy alone. PRODIGE-7 is the most recent trial comparing CRS-HIPEC with CRS in association with systemic chemotherapy [22]. The authors found no significant difference between groups in terms of OS and progression-free survival, concluding that the addition of oxaliplatin to HIPEC did not influence OS.

It is widely acknowledged that the completeness of cytoreduction is one of the key factors to prolonging survival. In our study, the group of patients undergoing CRS-HIPEC showed an almost fourfold longer median survival than those who had incomplete cytoreduction, a finding also confirmed by other authors [7, 9, 23, 24]. As the probability of achieving a complete macroscopic cytoreduction could be related to the experience of the surgeon [10], we believe that patients requiring CRS-HIPEC should be referred to specialized high-volume centers. This was also recommended in the most recent ESMO guidelines on the topic [11].

As shown in our study, PCI was a significant prognostic factor in patients who underwent CRS-HIPEC. In fact, this variable has been recognized as one of the most important prognostic factors in patients with PC from CRC [7, 23, 25–27]. Goéré et al. found that CRS-HIPEC was not associated with a survival benefit in patients with a PCI score of ≥ 17 when compared with palliative treatment [28]. We found that N2 patients with PCI > 6 had a significantly poorer prognosis with a median survival of 18 months. Taking into account this latter finding, which is very similar to results achieved with modern chemotherapy, we believe that CRS-HIPEC might not be indicated in N2 patients with PCI > 6. Larger prospective studies are required to confirm these findings and improve patients’ selection.

The limitations of our study are linked to its retrospective nature given that selection bias may have affected the entire cohort. Furthermore, the lack of perioperative data in the group of patients with incomplete CRS did not permit a proper comparison of short-term outcomes of surgery.

## Conclusions

CRS-HIPEC can greatly improve survival in CRC patient with PC. Complete cytoreduction (CC0) is required to achieve the best long-term results. Patients with high PCI (PCI > 6) and significant nodal involvement (N2) may not benefit from the procedure.

## Abbreviations

CRC: Colorectal cancer
CRS-HIPEC: Cytoreductive surgery plus hyperthermic intraperitoneal chemotherapy
DFS: Disease-free survival
DFS: Median disease-free survival
HR: Hazard ratios
OS: Overall survival
PC: Peritoneal carcinomatosis
PCI: Peritoneal cancer index
